# Detection of Counterfeit Perfumes by Using GC-MS Technique and Electronic Nose System Combined with Chemometric Tools

**DOI:** 10.3390/mi14030524

**Published:** 2023-02-24

**Authors:** Youssra Aghoutane, Mihai Brebu, Mohammed Moufid, Radu Ionescu, Benachir Bouchikhi, Nezha El Bari

**Affiliations:** 1 Biosensors and Nanotechnology Group, Department of Biology, Faculty of Sciences, Moulay Ismaïl University of Meknes, B.P. 11201, Zitoune, Meknes 50070, Morocco; 2 Sensor Electronic & Instrumentation Group, Department of Physics, Faculty of Sciences, Moulay Ismaïl University of Meknes, B.P. 11201, Zitoune, Meknes 50070, Morocco; 3 “Petru Poni” Institute of Macromolecular Chemistry, 700487 Iasi, Romania; 4 Institute of Veterinary Medicine and Animal Sciences, Estonian University of Life Sciences, 51006 Tartu, Estonia

**Keywords:** perfumes, counterfeit, volatile organic compounds, allergens, electronic nose, GC-MS

## Abstract

The Scientific Committee on Cosmetic and Non-Food Products has identified 26 compounds that may cause contact allergy in consumers when present in concentrations above certain legal thresholds in a product. Twenty-four of these compounds are volatiles and can be analyzed by gas chromatography-mass spectrometry (GC-MS) or electronic nose (e-nose) technologies. This manuscript first describes the use of the GC-MS approach to identify the main volatile compounds present in the original perfumes and their counterfeit samples. The second part of this work focusses on the ability of an e-nose system to discriminate between the original fragrances and their counterfeits. The analyses were carried out using the headspace of the aqueous solutions. GC-MS analysis revealed the identification of 10 allergens in the perfume samples, some of which were only found in the imitated fragrances. The e-nose system achieved a fair discrimination between most of the fragrances analyzed, with the counterfeit fragrances being clearly separated from the original perfumes. It is shown that associating the e-nose system to the appropriate classifier successfully solved the classification task. With Principal Component Analysis (PCA), the three first principal components represented 98.09% of the information in the database.

## 1. Introduction

Perfumes are among the most important and valuable products worldwide. These products consist of highly complex formulas derived from several natural sources, including flowers, leaves, roots, fruit barks, plant pigments, resins, and balsams, in combination with various chemical products [1]. 

Since original perfumes remain relatively more expensive, in recent years the high demand for cheaper perfumes has led to an explosion in the sale of counterfeit perfumes. Counterfeit perfumes often contain low quality ingredients in random concentrations. However, the obtained counterfeit perfumes generally smell similar to the original brands. As a result, there is an increasing overproduction of counterfeit perfumes around the world [2,3]. Imitation perfumes are very detrimental to the perfume industry in general, and to perfume consumers in particular. On the one hand, the manufacturers of imitation perfumes harm the cosmetics industry due to the economic losses they cause. On the other hand, because the correct concentrations are not respected, counterfeits are very harmful to the health of users in the long run. These chemical compounds are less tolerated by users and can cause allergic skin reactions, respiratory, neurological, and nasal symptoms [4]. Therefore, determining the safety of fragrance ingredients is a top priority.

Conventionally, traditional analytical methods have been used to analyze the volatile organic compounds emitted by these complex fragrances, such as gas chromatography-mass spectrometry (GC-MS) [5,6,7,8,9]. More recently, direct infusion electrospray ionization (ESI-MS) [10], room temperature sonic spray ionization (EASI-MS) [11], and extractive electrospray mass spectrometry (EESI-MS) [12] have been applied to classify fragrances and identify fraudulent samples. In these studies, researchers were able to identify counterfeit fragrances based on the differences in mass spectra signatures between the original and counterfeit samples, without the need for detailed chemical composition analysis.

However, in situations where time and cost are critical, these methods prove to be excessively slow and costly. Recently, different approaches have been introduced for the analysis of volatile organic compounds emitted by fragrances, based on the use of artificial electronic olfactory systems called electronic nose (e-nose) [13,14,15,16,17,18]. These systems present important advantages, including simplicity, affordability, non-destructive measurements, and ability to be used as a portable device.

E-nose instruments have been applied in many areas of science and industry, for example in the biomedical field diagnosis [19], food safety [20,21,22], environmental pollution monitoring [23], and pharmaceutics applications [24]. E-nose systems have also been used for the classification of cosmetics, essential oils, and perfumes [13,14,25]. An e-nose system based on 18 metal oxide semiconductor gas sensors has been successfully used to classify three Yves Saint Laurent perfumes [18]. Similarly, two custom-built prototype e-noses equipped with six and twelve solid-state gas sensors from Figaro, respectively, were used to distinguish between genuine and fake perfume samples from Dior-Fahrenheit, Eisenberg-J’ose, Yves Saint Laurent-La nuit de L’homme, Loewe, and Spice Bomb [26]. Metal oxide gas sensors have also been used in other detection devices, applying odor detection mechanisms [27].

The e-nose is based on a network of cross-reactive chemical gas sensors combined with a pattern recognition algorithm for data classification [14,15,16]. Among the most frequently used sensors in e-nose device are the Figaro TGS solid-state gas sensors. Some of the important features of these sensors are fast response time, good recovery from sample measurement, and long life [28,29]. Moreover, several experiments have shown that metal oxide gas sensors are the most suitable chemical sensors for long term application, of more than one year of continuous operation [30].

In the present study, we tested the ability of an own-developed electronic nose system, consisting of an array of 6 gas sensors (TGS-type) [31], in order to analyze the VOCs emanating from both original fragrances and their counterparts. Moreover, the same samples were also analyzed by GC-MS methods to identify allergens in all fragrance samples. The study was performed on perfume samples referred to as A.O, B.O, C.O, D.O, and E.O, while their imitations were referred to as A.I, B.I, C.I, D.I, and E.I, for confidentiality reasons. The possibility of distinguishing the five authentic perfumes from their counterfeits was demonstrated by using the e-nose device based on TGS-type semiconductor gas sensors.

## 2. Materials and Methods

### 2.1. Perfumes

Five original fragrances of commercial brand perfumes labelled as “eau de toilette” were purchased from a popular international perfumery that enjoys a great reputation. The counterfeit samples used in this study were purchased on free markets, and their counterfeit nature was revealed by the supplier without proof that s/he is an authorized provider. The packaging of these perfumes was not marked. For confidentiality reasons, the analyzed samples were labelled with letters from A. to E., followed by the letter O for evoking the original fragrances and by letter I for evoking their imitations, respectively. A.O is a feminine aldehydic floral fragrance, while B.O is a sensual feminine floral perfume with a sparkling ginger base. The other three (C.O, D.O, and E.O) are masculine fragrances based on anise, Italian mandarin, cedar wood, and musk notes. These fragrances were selected because they are among the most famous and onerous perfumes in the world and are widely used by women and men.

### 2.2. Gas Chromatography-Mass Spectrometry (GC-MS) Analysis

Among the analytical methods dedicated to measuring volatile organic compounds, we opted for the most used technic, namely Gas Chromatography-Mass Spectrometry (GC-MS). The principle of the gas chromatography method is the separation of the mixture components, while the aim of mass spectrometry is to detect and identify the separated components.

GC-MS analysis of all samples was performed with a 6890 Agilent Technologies gas chromatograph coupled to a 5975 Inert XL Agilent Technologies mass detector working at 70 eV ionization energy. Chromatographic separation was performed on a Teknokroma TR-520232 capillary column of 30 m length and 0.25 mm internal diameter, coated with a 5% diphenyl/95% dimethyl polysiloxane film of 0.25 µm thickness. Ultrapure 5.5 grade He was used as carrier gas at 0.5 mL/min flow rate. The temperature program for the GC column was as follows: initial temperature of 60 °C, heating rate of 4 °C/min up to 220 °C, then of 20 °C/min up to 320 °C, and finally isothermal at 320 °C for 1 min. Volumes of 0.1 µL perfume samples were directly injected into the GC, without any pre-treatment, at a 50:1 split ratio, in the inlet port heated at 230 °C. The transfer line between the GC column and the MS detector was heated at 280 °C.

Compound identification from the acquired chromatographic peaks was achieved based on National Institute of Standards and Technology (NIST 14) reference library database, Kovats retention index, and cross-validation among samples. Compounds with a quality of recognition below 90%, which were identified as having abnormal retention times, or that were impossible to cross-validate, were disregarded. In the case of interference conditions such as improper chromatographic separation of similar compounds, identification was performed based on single ion chromatograms of the main mass fragments in the MS spectra. To avoid interference with the background noise, only the chromatographic peaks with area above 0.5% from the total area of the chromatogram were considered.

The particular GC conditions were selected based on previous experience on GC analysis of essential oils. The conditions were optimized in terms of temperature program and carrier gas flow (to ensure proper separation of compounds on the entire range, from low to high molecular weight), temperature of inlet (to ensure total volatilization of compounds of interest), sample volume, and split ratio (to avoid saturation of the detector). Since essential oils, as well as perfumes, have a complex composition (usually more than 100 distinct compounds), validation based on standard chemicals could not be performed for each particular compound.

### 2.3. E-Nose Setup and Measurements

**Figure 1:**
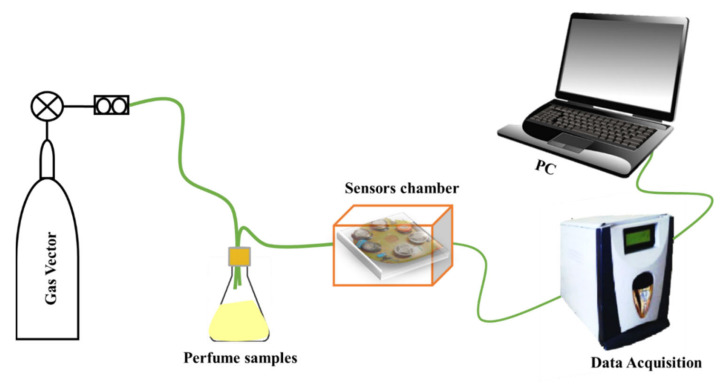
E-nose experimental set-up used for the sensing measurements.

The e-nose system employed in this study was based on six semiconductor chemical gas sensors purchased from Figaro Engineering, Inc. (Osaka, Japan) (see Table S1) [31,32]. It consisted of three distinct parts: the sampling unit, the sensor chamber, and the data acquisition system (Figure 1).

To perform the sample measurements with the e-nose, 100 µL of each fragrance was placed in a conical 1 mL bottle with two small holes drilled in the lid, as shown in Figure 1. The reference gas bottle and the sensor chamber were connected by means of a PTFE tube with an inner diameter of 40 mm and an outer diameter of 50 mm. Synthetic dry air, employed as reference gas (flushed for 8 min at 100 mL/min flow rate through the measurement setup), conveyed the volatile organic compounds from the fragrance headspace formed inside the conical flask to the sensor array. After sample measurement, the e-nose was exposed to synthetic dry air for 10 min at a flow rate of 100 mL/min. The purpose of this step was to purge the sensor test chamber and permit the gas sensors to return to their baseline conditions. 

To perform the sample measurements with the e-nose, 100 µL of each fragrance was placed in a conical 1 mL bottle with two small holes drilled in the lid. In addition, the reference gas bottle and the sensor chamber were connected by means of PTFE tube with an inner diameter of 40 mm and an outer diameter of 50 mm.

The conical flask was cleaned with distilled water and synthetic dry air for 100 s before and after each sample measurement. This operation removed all traces of the previously measured perfume. The measurement of each sample was repeated 3 times in order to ensure repeatability of the measurements.

### 2.4. Data Analysis

Following e-nose measurements, one feature was extracted from the response of each gas sensor of the array, which is the difference between the stabilized sensor conductance after fragrance exposure (G_s_) and the initial baseline conductance (G_0_):

ΔG = (G_s_ − G_0_) (1)

The radar plot was at first used as graphical representation. The purpose of this graph was to observe the similarities or differences between the representative volatile organic compounds identified by the GC-MS method, collected from the original perfumes and their imitations, as well as between the overall responses of the e-nose to these fragrances. The radar plot is strongly recommended before applying a statistical or pattern recognition method, as it frequently anticipates the classification of data groups.

Following the extraction stage, the obtained data were normalized and centralized, and then examined using data processing methods such as principal component analysis (PCA) and discriminant function analysis (DFA) (also known as linear discriminant analysis (LDA) or normal discriminant analysis (NDA)) to improve visualization and comprehension of the studied samples.

PCA is a powerful unsupervised linear pattern recognition technique that has proven to be effective in easily visualizing the maximum amount of information in a dataset [33]. It decomposes the primary data matrix by projecting the multidimensional data into a new coordinate base. This is formed by orthogonal directions with maximum data variation. In the present study, PCA was employed as an exploratory data analysis approach in order to find patterns of the available data.

The DFA method is a frequently used supervised pattern recognition algorithm in data classification [34]. It is based on the determination of discriminant functions, which maximizes the variance between classes and minimizes the intra-class variance ratio. The classes need to be a priori defined. In the present study, two classes were totally defined, corresponding to the original perfumes and their counterfeit fragrance, respectively.

## 3. Results and Discussion

### 3.1. GC-MS Results

The quantitative analysis of the original and imitation perfumes was performed by GC-MS. The goal was to identify counterfeit additions that could be detrimental to well-being and health.

**Figure 2:**
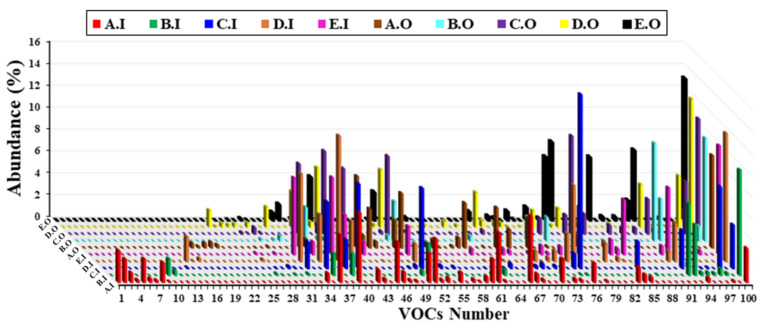
Histogram performed employing the GC-MS results of the volatile organic compounds detected in the different fragrances of the original and imitation perfumes.

The indicative names of the main one hundred volatile organic compounds identified by the GC-MS method are presented in Table S2. The GC-MS analysis revealed differences between the composition of the original perfumes and their imitations, as observed in Figure 2, which represents the histogram of the volatile organic compounds. We can notice from this histogram the presence of specific volatile organic compounds (VOCs numbered from 1 to 17) in the imitation perfume samples. Moreover, the VOCs numbered from 18 to 28 were only found in the original perfumes. On the other hand, the other volatile organic compounds were detected in different concentrations in both perfumes. In fact, some of them are common to all fragrances of the perfumes.

**Figure 3:**
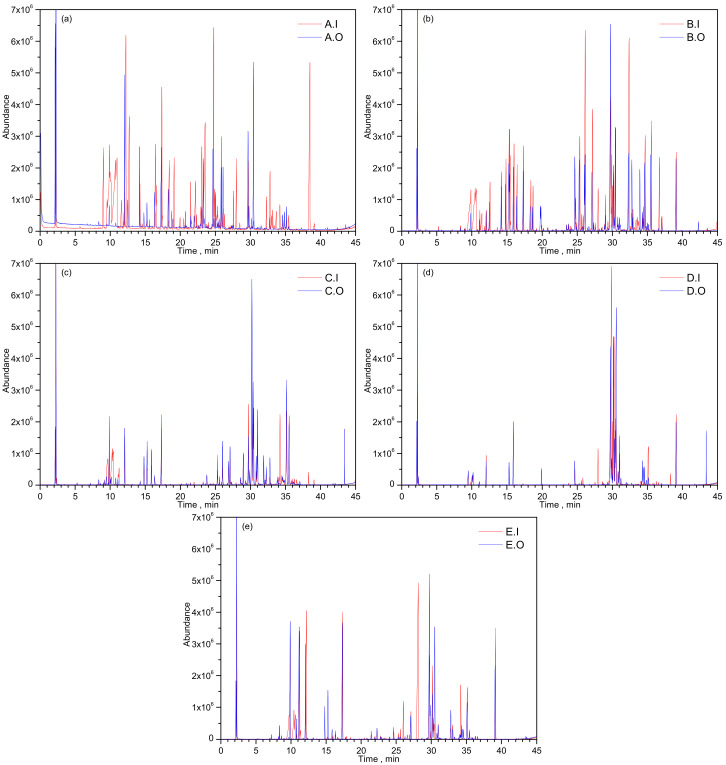
Total Ion Current Chromatogram (TIC) of: (**a**) A.O and A.I perfume, (**b**) B.O and B.I perfume, (**c**) C.O and C.I perfume, (**d**) D.O and D.I perfume, and (**e**) E.O and E.I perfume.

Figure 3 describes the total ion current chromatogram (TIC) obtained following samples analysis by the GC-MS method. According to these chromatograms, the number of volatile organic compounds released by the original fragrances is lower than that of their imitations. This result demonstrates that there are more volatile organic compounds in the fake perfumes than in the original ones. Many of these additives are powerful allergens that are harmful to health.

As further disclosed in Table 1, the most important allergens detected in the counterfeit perfumes were benzyl alcohol (A.I), hydroxycitronellal (A.I), benzoic acid 2-hydroxy-ethyl ester (D.I), linalool (E.I), D-limonene (all fake perfumes), citronellol (A.I, B.I, C.I and E.I), geraniol (E) (A.I), coumarin (A.I, B.I, C.I and E.I), isoeugenol (C.I), and benzyl benzoate (A.I, D.I and E.I). Some of these allergens were also found in the original fragrances, such as linalool (A.O and D.O), limonene (A.O and E.O), citronellol (all original perfumes), geraniol (E) (A.O), coumarin (A.O, B.O, C.O and D.O), isoeugenol (A.O and C.O), and benzyl benzoate (A.O and B.O).

For two of these allergens, citronellol and coumarin, their concentrations were similar in both original and counterfeit fragrances, but this was not the general rule. Some allergens were only contained by counterfeit fragrances, such as benzoic acid 2-hydroxy-ethyl ester, which was found in D.I at 0.1% concentration, or the two strong allergens benzyl alcohol and hydroxycitronellal that were only found in A.I, at 2.17% and 2.18% concentration, respectively. Their concentrations highly exceeded the standard limits established by the European Cosmetics Directive for these two compounds, which are set at 0.001% in non-rinsed and at 0.01% in rinsed products, respectively [35,36,37,38]. This finding shows that A.I can be extremely dangerous for the health of the users.

Another observation was that, among all perfumes, the A fragrances contained the highest number of the allergens, and generally in much higher levels than all the other perfumes (e.g., citronellol, coumarin and benzyl benzoate), while geraniol (E) was only found in the A fragrances.

Linalool was found in the counterfeit fragrance E.I, at 1.21% concentration, but also in two original perfumes, A.O and D.O, at high concentrations of 6.6% and 6.04%, respectively.

As observed, all the counterfeit perfumes contained different additives and allergens in comparison with the originals, with generally higher concentrations. Even though the original fragrances also contain some allergens, it should be noted that these allergens are always indicated on their label, so that the consumer has the possibility to be informed about their presence. However, this is not the case for the counterfeit perfumes, which may contain many high concentration allergens without being declared on the perfume label.

**Figure 4:**
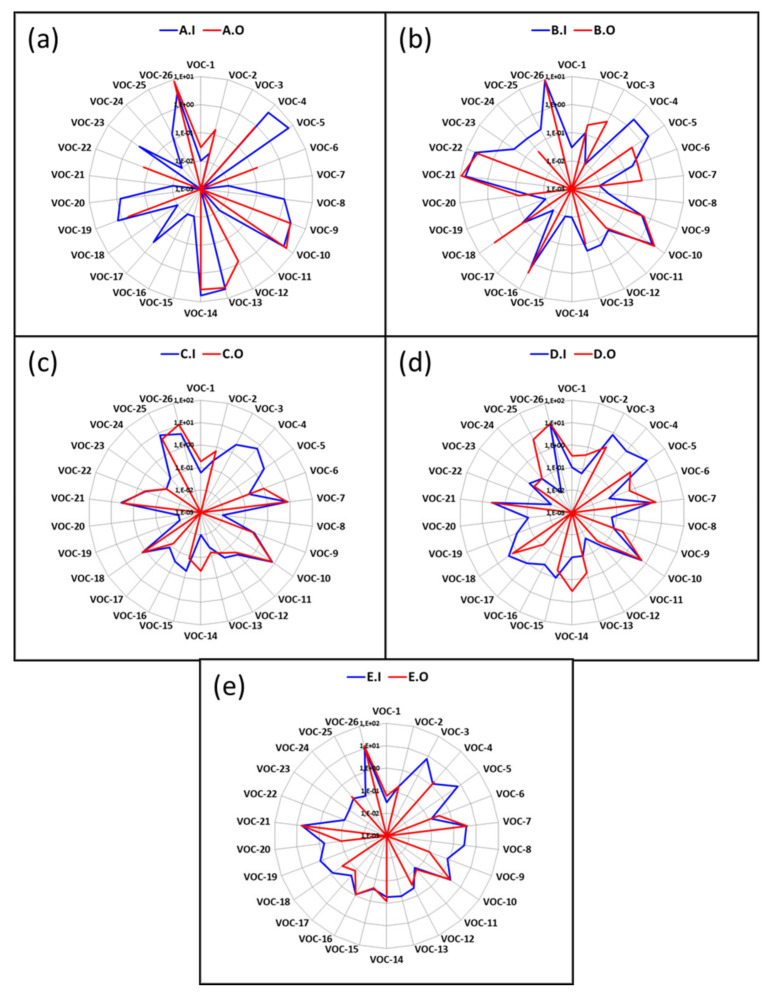
Radar plot representing common VOCs detected by GC-MS from original perfumes and their counterfeits: (**a**) A.O and A.I perfume, (**b**) B.O and B.I perfume, (**c**) C.O and C.I perfume, (**d**) D.O and D.I perfume, and (**e**) E.O and E.I perfume.

Figure 4 shows the radar plot of the volatile organic compounds found simultaneously in the original perfumes and their counterfeits, which are among the common compounds responsible for the specific quality and smell of a perfume. It can be seen from this figure that the fingerprints of the original perfumes are different and smaller than those emitted by their imitations. This is due to the many different compositions used by counterfeiters when trying to reproduce the original perfumes.

### 3.2. E-Nose Results

#### 3.2.1. Sensor Responses

The evolution of the conductance signals (G(t)) generated by the gas sensor array exposed to the samples of the original perfume A.O and its counterfeit A.I is depicted in Figure S1. The conductance of the six sensors increased upon exposure to the perfume samples (0–140 s time interval), followed by a decrease for the recuperation of their baseline conditions upon the injection of synthetic dry air for purging purposes (140 s time point), while slight differences in the conductance responses were observed between the original and counterfeit perfume. The highest response was observed for the TGS 825 sensor.

#### 3.2.2. Samples Classification

**Figure 5:**
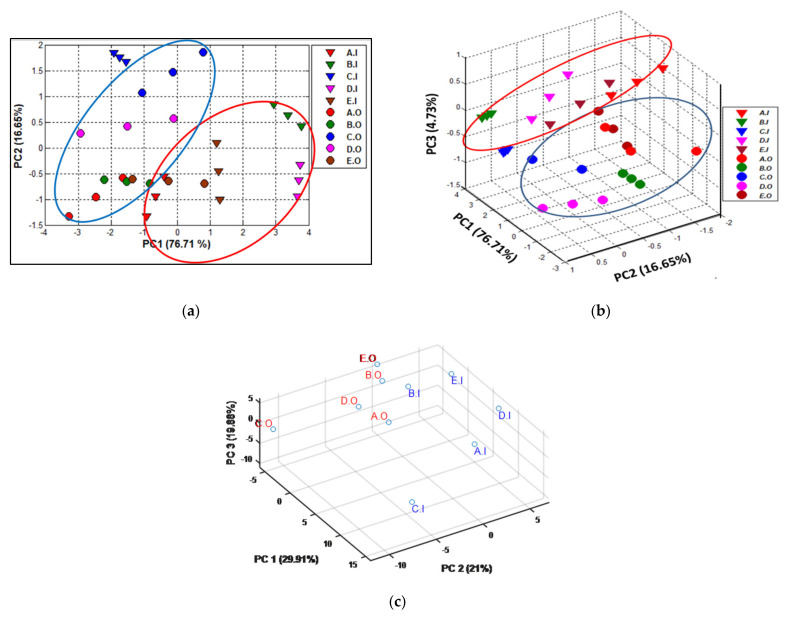
Two-dimensional PCA plot performed on original fragrances and their imitations based on e-nose results (**a**); Three-dimensional PCA plot performed on original fragrances and their imitations based on e-nose results (**b**); (**c**) GC-MS compositional analysis.

Figure 5 shows samples projection on the PCA graph plotted using the first two (Figure 5a) and three (Figure 5b) main principal components, which accounted for 93.96% and 98.09% of the total data variance, respectively. A fair discrimination of the samples was obtained using the (2D) PCA plot, with two classification errors of the perfume samples C.I and E.O. By employing the third PC, an improved discrimination was achieved between the original and counterfeit samples, with the exception of one sample of E.O that was projected close to the counterfeit group, with the third component being able to provide additional important information about the data. The PCA plot shows a slight overlapping of the samples of each group related to original perfume and the counterfeit samples, respectively. Original samples were closer grouped one to each other, due to reproducible sets of polar components (chemical signatures of each fragrance). In contrast, counterfeit samples were wider dispersed, due to the diversity of counterfeiting procedures and compositions.

From this figure, it can be noted that perfumes and their imitations can be perfectly distinguished by the e-nose. A good classification based on PCA was also obtained from the chemical composition of the fragrances determined by the GC-MS method listed in Table S2, Figure 5c. Although the C samples were separately placed from the other fragrances, a good discrimination was achieved between the original and the counterfeited one.

**Figure 6:**
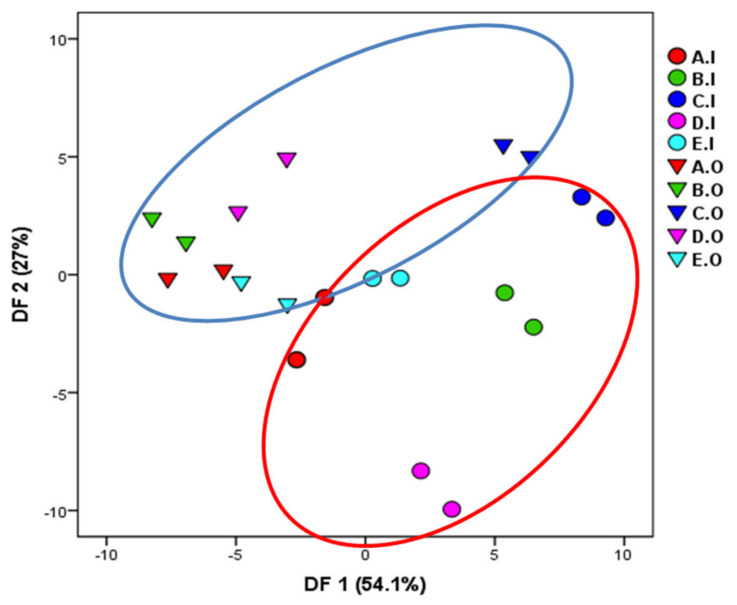
Plot of the discriminant function analysis (DFA) performed on original fragrances and their counterfeits based on e-nose results.

The dataset gathered from e-nose was also treated using DFA. As DFA is a supervised pattern recognition algorithm, only two samples per fragrance were used to build the classification model in order to avoid overfitting problems. Figure 6 shows the DFA plot of all analyzed fragrances. All samples except E.O. were well-separated from each other, confirming the PCA results. 

The results obtained in this study confirmed that data treatment by using DFA permits to discriminate well between the original fragrances and their imitations.

## 4. Conclusions

Counterfeit perfumes cause consumer deception and health problems for users. In this study, the analysis of volatile organic compounds was used to differentiate between the original perfumes and their imitations. GC-MS was first used to identify the main volatile organic compounds present in all fragrance samples. It was found that the imitations contained significantly more volatile compounds than the original fragrances, while some allergens were identified in much higher concentrations or only in the imitated fragrances.

An e-nose system based on six Figaro solid-state gas sensors was then used to differentiate the odors of the different fragrances with satisfactory results. The classification models constructed from the responses to the sensor data successfully classified the original fragrances from their imitations employing both PCA and DFA pattern recognition algorithms. This classification was based on the differences in chemical composition of the samples.

The results obtained in this study reinforce the fact that the e-nose approach holds good potential for counterfeit perfumes identification. Based on its great simplicity, affordable price, efficiency, and rapidity, the e-nose technology could become a reference standard in the future for conveniently determining the authenticity and quality of products in the perfume and other cosmetic industries. E-nose measurements provide other important features, such as the possibility to analyze liquid samples without pre-treatment, easy sample handling without the need for gas syringe injection, portability, and easy selection and exchange of sensors. Therefore, it may offer the possibility of being introduced as a routine method in a perfume quality control laboratory.

## Figures and Tables

**Table 1:** Harmful allergens identified in the perfume samples.

Allergen	IUPAC Name	Retention Index (Ri)	Perfume Sample	Compound Concentration (%)	EU Standard Limits (%)
Benzyl alcohol	-	1036	A.I	2.17	0.001 (non-rinsed cosmetic products) 0.01 (rinsed cosmetic products)
Hydroxycitronellal	7-Hydroxy-3,7-dimethyloctanal	1231	A.I	2.18	0.001 (non-rinsed cosmetic products) 0.01 (rinsed cosmetic products)
Benzoic acid 2-hydroxy-ethyl ester	2-Hydroxyethyl benzoate	1380	D.I	0.1	2.0
Linalool	3,7-Dimethyl-1,6-octadien-3-ol	1082	A.I D.I E.I	6.6 6.04 1.21	0.001 (non-rinsed cosmetic products) 0.01 (rinsed cosmetic products)
Limonene	(R)-p-Mentha-1,8-diene	1018	A.I B.I C.I D.I E.I A.O E.O	4.36 2.03 6.16 4.34 1.23 0.85 1.5	0.001 (non-rinsed cosmetic products) 0.01 (rinsed cosmetic products)
Citronellol	3,7-Dimethyl-6-octen-1-ol	1179	A.I B.I C.I E.I A.O B.O C.O D.O E.O	2.72 0.46 0.35 0.08 2.57 0.53 0.3 0.25 0.11	0.001 (non-rinsed cosmetic products) 0.01 (rinsed cosmetic products)
Geraniol(E)	3,7-Dimethyl-2,6- octadien-1-ol	1352	A.I A.O	0.33 0.09	0.001 (non-rinsed cosmetic products) 0.01 (rinsed cosmetic products)
Coumarin	1-Benzopyran-2-one	1374	A.I B.I C.I E.I A.O B.O C.O D.O	4.59 0.18 0.04 0.58 4.15 0.1 0.07 0.59	0.003
Isoeugenol	2-Methoxy-4-(prop-1-enyl)phenol	1410	C.I A.O C.O	0.08 0.11 0.12	0.001 (non-rinsed cosmetic products) 0.01 (rinsed cosmetic products)
Benzyl benzoate	Benzyl benzoate	1733	A.I D.I A.O B.O	1.4 0.4 1.41 0.57	0.001 (non-rinsed cosmetic products) 0.01 (rinsed cosmetic products)

## Data Availability

The data presented in this study are available on request from the corresponding author. The data are not publicly available due to confidentiality reasons.

## Supplementary Materials

The following supporting information can be downloaded at: https://www.mdpi.com/article/10.3390/mi14030524/s1 Table S1: Sensitivities of the TGS sensors used in the electronic nose unit; Table S2: Indicative names of the main volatile compounds in the perfume samples identified by GC-MS technique; Figure S1: Dynamic response of the six gas sensors array in the form of conductance when exposed to: (a) sample of original (A.O) perfume, (b) sample of counterfeit (A.I) perfume.
