# Supplementary material for: Detection of Counterfeit Perfumes by Using GC-MS Technique and Electronic Nose System Combined with Chemometric Tools
Source: Micromachines (Basel). 2023 Feb 24;14(3):524. doi: 10.3390/mi14030524 (PMC10052770; doi:10.3390/mi14030524)

Supplementary Materials

Detection of counterfeit perfumes by using GC-MS technique and electronic nose system combined with chemometric tools

Youssra Aghoutane [1,2], Mihai Brebu [3], Mohammed Moufid [1,2], Radu Ionescu [4], Benachir Bouchikhi [2] and Nezha El Bari [1,*]

[1] Biosensors and Nanotechnology Group, Department of Biology, Faculty of Sciences, Moulay Ismaïl University of Meknes, B.P. 11201, Zitoune, Meknes 50070, Morocco; n_elbari@hotmail.com
[4] Sensor Electronic & Instrumentation Group, Department of Physics, Faculty of Sciences, Moulay Ismaïl University of Meknes, B.P. 11201, Zitoune, Meknes 50070, Morocco
[3] "Petru Poni" Institute of Macromolecular Chemistry, 700487 Iasi, Romania.
[4] Institute of Veterinary Medicine and Animal Sciences, Estonian University of Life Sciences, 51006 Tartu, Estonia.
* Correspondence: n_elbari@hotmail.com; Tel.: +212 661358432 / +212 535 53 88 70 1

Table S1. Sensitivities of the TGS sensors used in the electronic nose unit

Sensor Name	Target gases
**TGS 815**	High sensitivity and high selectivity to methane, propane, and butane
**TGS 821**	Highly sensitive to organic solvent vapors and a wide variety of combustible gases such as carbon monoxide, and hydrogen
**TGS 822**	Organic solvents
**TGS 824**	NH_3
**TGS 825**	H_2S, Ethylene
**TGS 842**	Domestic gas alarm, alcohol vapors, and methane

Table. S2 Indicative names of the main volatile compounds in the perfume samples identified by GC-MS technique.

No.	Compounds	No.	Compounds	No.	Compounds
**1**	Ethanol, 2-(2-ethoxyethoxy)-	34	Benzene, 1-methoxy-4-methyl (p-Methylanisole)	67	3 trans-β-Ionone
**2**	Benzyl alcohol	35	Cyclosia (Hydroxycitronellal)	68	Benzene, 1,2-dimethoxy-4-(1-propenyl)- (Isoeugenol methyl ether)
**3**	Benzenepropanol	36	Limonene	69	Cashmeran
**4**	Propanoic acid, phenylmethyl ester	37	Limonene	70	Butylated hydroxytoluene (Antioxidant 29)
**5**	Hydroxycitronellal	38	Eucalyptol	71	1-Penten-3-one, 1-(2,6,6-trimethyl-2-cyclohexen-1-yl) (α-N-Methyl ionone)
**6**	10-Undecenal	39	Dipropylene glycol isomer/derivative	72	Lilial
**7**	2,6-Octadiene, 2,6-dimethyl-	40	Terpinene	73	Benzoic acid, 2-hydroxy-, pentyl ester (Amyl salicylate)
**8**	Benzaldehyde, 3-hydroxy-4-methoxy-(vanillin)	41	Dihydromyrcenol	74	Butylated hydroxytoluene
**9**	α-Gurjunene	42	cis-Linalyl oxide	75	Helional
**10**	Helional	43	Benzoic acid, methyl ester	76	Diethyl phthalate
**11**	Isoaromadendrene epoxide	44	1 β-Linalool	77	Guaiol
**12**	Methyl dihydrojasmonat	45	Phenylethyl alcohol	78	Cedrol
**13**	2-Butanol, 3,3'-oxybis	46	Benzyl acetate	79	Ethyl citrate
**14**	Benzenemethanol, α-methyl-, acetate	47	1,2-Dihydropyridine, 1-(1-oxobutyl)	80	Methyl dihydrojasmonate
**15**	Dipropylene glycol isomer/derivative	48	Decanal	81	Atraric acid
**16**	Benzoic acid, 2-hydroxy-, ethyl ester	49	Linalyl formate	82	Ambrox
**17**	Allyl (isopentyloxy)acetate	50	β-Citronellol	83	Benzyl benzoate
**18**	Phenylallyl alcohol (Cinnamyl alcohol)	51	Linalyl anthranilate	84	Octanal, 2-(phenylmethylene) (α-Hexylcinnamaldehyde)
**19**	Benzene, 2-(1,1-dimethylethyl)-1,4-dimethoxy	52	Piperonal	85	Isopropyl myristate
**20**	2-Propen-1-ol, 3-phenyl-, acetate	53	Phenol, 2-methoxy-3-(2-propenyl)	86	9-Decen-1-ol
**21**	β-Cubebene	54	2,6-Octadien-1-ol, 3,7-dimethyl-, acetate, (E)- (Geraniol acetate)	87	Musk ambrette, natural
**22**	α-Farnesene	55	Geraniol acetate	88	Lycopersilure
**23**	2-Butanol, 3,3'-oxybis	56	Vanillin	89	11-Tetradecen-1-ol, (E)
**24**	Ethyl linalool	57	Dodecanal	90	11-Dodecenol
**25**	Cyclohexene, 1-methyl-4-(1-methylethylidene)-	58	α-Cedrene	91	Muskalactone
**26**	α-Ionone, 6-methyl-	59	Caryophyllene	92	Galoxolide

**27**	2-Propanol, 2-methyl-	60	α-Ionone	93	Benzoic acid, 2-hydroxy-, phenylmethyl ester (Benzyl salicylate)
**28**	α-Pinene	61	Coumarin	94	Polycyclic musk
**29**	Menthene	62	trans-Isoeugenol, (E)	95	7-Acetyl-6-ethyl-1,1,4,4-tetramethyltetralin
**30**	ß -Pinene	63	Ethyl vanillin	96	Cyclopenta[g]-2-benzopyran, 1,3,4,6,7,8-hexahydro-4,6,6,7,8,8-hexamethyl-
**31**	ß--Myrcene	64	Isoeugenol methyl ether	97	Acetophenone, 4'-tert-butyl-2',6'-dimethyl-3',5'-dinitro- (Musk 36A)
**32**	Hexylacetate	65	α Isomethyl ionone (α-Cetone)	98	Ethylene brassylate (Musk T)
**33**	Dipropylene glycol isomer/derivative	66	3-Penten-2-one, 4-(2,6,6-trimethyl-2-cyclohexen-1-yl)	99	2-Propenoic acid, 3-(4-methoxyphenyl)-, 2-ethylhexyl ester (Parsol MCX)
			100 Ethanol		

Figure S1.

Dynamic response of the six gas sensor array in the form of conductance when exposed to: (a) sample of original (A.O) perfume, (b) sample of counterfeit (A.I) perfume

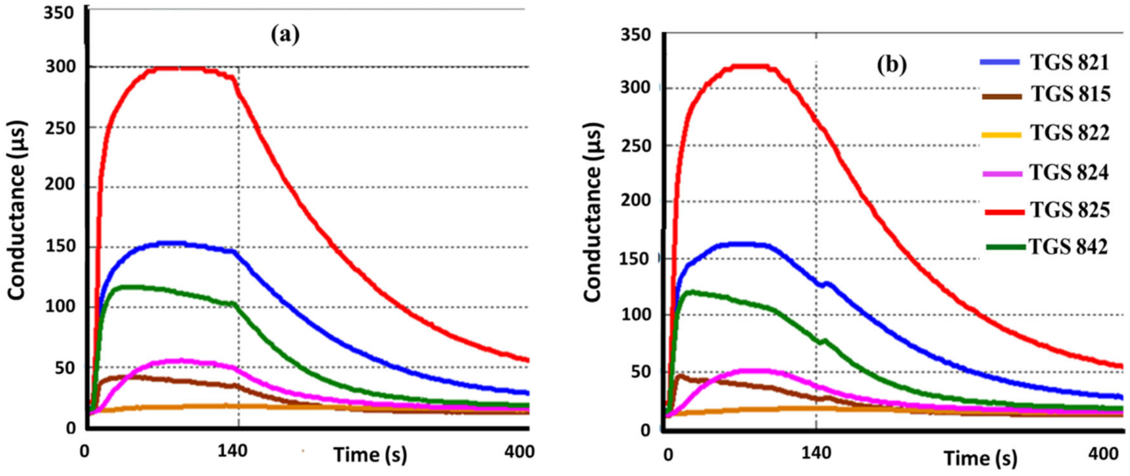

Supplement: Supplementary file 1 [file micromachines-14-00524-s001.zip › micromachines-2189447-supplementary.pdf]
